# Self-Initiated Dietary Adjustments Alter Microbiota Abundances: Implications for Perceived Health

**DOI:** 10.3390/nu16203544

**Published:** 2024-10-18

**Authors:** Anouk Willems, Martina Sura-de Jong, Eline Klaassens, Bartholomeus van den Bogert, André van Beek, Gertjan van Dijk

**Affiliations:** 1 Applied Research Centre Food & Dairy, Van Hall Larenstein University of Applied Sciences, 8934 CJ Leeuwarden, The Netherlands; 2 Groningen Institute for Evolutionary Life Sciences—Neurobiology, University of Groningen, 9474 AG Groningen, The Netherlands; 3 Product Development Department, BaseClear B.V., 2333 BE Leiden, The Netherlands; 4 MyMicroZoo, 2333 BE Leiden, The Netherlands; 5 Department of Endocrinology, University Medical Center Groningen, Postbus 30001, 9700 RB Groningen, The Netherlands

**Keywords:** dietary patterns, gut microbiota, non-communicable disease, mental symptoms, somatic symptoms

## Abstract

**Background/Objectives:** Personalized and self-initiated dietary adjustments have been shown to alleviate mental and somatic complaints. Here, we investigated the potential role of gut microbiome alterations underlying these effects. **Methods:** For this purpose, participants (n = 185) underwent a four-week self-initiated dietary intervention and filled out weekly questionnaires on their dietary intake, somatic and mental symptoms, and physical activity. **Results:** Overall, the participants lost weight, had alleviated mental and somatic complaints, reduced their total caloric and percentual carbohydrate intake, and ate less processed, party-type, and traditional Dutch food items, but ate more Pescatarian type food items, while keeping their fiber intake unaltered. Baseline and endpoint gut microbiota analyses using 16S rRNA gene sequencing revealed an overall increase in *Gemmiger formicilis* and reductions in *Peptostreptococcaceae* and *Ruminococcus bromii* over the four-week dietary intervention. While these bacterial alterations were considered to be beneficial for the host, they were not individually correlated with alterations in, or endpoint levels of, somatic and/or mental complaints. Instead, individual increases in *Ruminococcus bicirculans* (a well-known utilizer of plant cell wall polysaccharides) were strongly correlated with reductions in mental complaints, even though overall *R. bicirculans* remained unaltered over the course of the four-week self-initiated dierary intervention. **Conclusions:** Our results suggest that overall altered versus individually correlated microbiota abundances and their relations with host health characteristics over the course of a self-chosen dietary intervention may represent different levels of regulation, which remain to be further untangled.

## 1. Introduction

The relation between the gut microbiota and the host has become a field of intense research. Interactions between the microbiota and the host are manifold, and compositional shifts in pathogenic and health-promoting commensal microbes have been implicated in a multitude of metabolic and non-communicable diseases (NCDs) [1,2,3,4]. As for specific microbes, several taxonomic markers have also been proposed to be related to health. For instance, an increased Firmicutes to Bacteroidetes ratio, which are the most prominent phyla in the gut, is often related to the incidence of obesity [5], although this relation has also been debated [6,7]. The Shannon diversity index, a frequently used measure of alpha diversity, has been found to be positively correlated with human health in epidemiological studies, and has also been shown to be reduced in individuals with obesity [8]. Methods that influence the gut microbiota composition may be a useful avenue for the treatment and prevention of these diseases [9]. Indeed, the gut microbiota composition has been recognized as a predictive biomarker for the success of body weight loss, which is an important risk factor for the development of NCDs [10]. As such, the manipulation of dietary intake has been suggested as one of the greatest and rapid modulators of the gut microbiota composition [11,12,13]. Although the consequences of various dietary patterns on human health are well known [14,15], there appear, however, large differences in how individuals respond to various diets [12,16]. Likewise, the composition of each individual’s gut microbiota appears to be quite unique too [17,18], and dietary intervention studies often show very heterogeneous and complex patterns of microbiota changes on a group level [19]. This calls for studies taking into account the individual differences in dietary intake at baseline and during and after interventions, and the correlated changes in markers for NCD risk, as well as correlated changes in the gut microbiota composition.

We have recently reported that a freely chosen and self-initiated dietary intervention leading to a spectrum of dietary patterns (and related macronutrient changes) is capable of alleviating NCD risk markers, such as mental and somatic symptoms [20,21], and reducing BMI and body weight [22]. In the current study, we aim to investigate whether and how the gut microbiota composition could be related to any of the changes in the aforementioned parameters of our study. For this purpose, the fecal microbiota composition was determined in a subset of individuals from our prior study, before and at the end of the freely chosen and self-initiated dietary change, and its relation with health outcomes was examined. Rather than focusing on the putative effects of specific diets on certain health outcomes, our study contributes to the rising research topic of precision nutrition tailored towards individuals as a means for treatment, reductions in preclinical symptoms, and the prevention of NCD’s [20,21,23,24,25,26,27].

## 2. Materials and Methods

### 2.1. Study Design

The study design and methods have previously been published and are briefly described here [22]. This study was a four-week dietary intervention in men and women aged ≥18 years, who were recruited from the general Dutch population using online advertisements (www.etenvoordewetenschap.nl, accessed on 27 August 2024), media (radio and newspaper) interviews, press releases, and informative presentations. The inclusion criteria were the willingness to change habitual dietary patterns, to complete the consent form, and to provide fecal samples. No exclusion criteria were applied. Participants were recruited between October 2018 and January 2020. Informed consent was obtained from all participants.

The participants were asked to commit to a freely chosen self-imposed dietary regime that they thought would promote their health and that they could maintain for a period of at least four weeks. Some examples of dietary patterns were offered to the participants. The changes could range from the adoption of an entirely new dietary pattern to the removal or addition of single food items. The results presented in this article were obtained from a subset of participants from our previous study [22], from whom we collected fecal samples at baseline and immediately after four weeks of intervention, in order to determine (changes in) their fecal microbiota composition.

This trial is registered by the medical ethical committee of the University Medical Centre Groningen in the Netherlands with number 2018/384 and was exempted from ethical approval.

### 2.2. Questionnaires

As previously described, demographic data, including age and sex, diagnosed disease, and medication use were obtained at baseline [22]. The participants filled out online questionnaires at baseline and weekly from the start of the intervention on their mental and somatic symptoms, dietary intake, and physical activity. Self-reported measures of body weight and waist circumference were gathered at the same time points. Mental symptoms were measured with the Depression, Anxiety, Stress Scales (DASS) [28,29]. Somatic symptoms were measured with the Patient Health Questionnaire (PHQ-15) [30]. Dietary intake was assessed with a food frequency questionnaire (FFQ), which measured the intake of 23 different food categories in portions per day or per week [22]. Physical activity was assessed with the Physical Activity Scale 2 (PAS-2), which measured physical activity in metabolic activity of task (Met) scores per 24 h [31].

### 2.3. Dietary Intake

Principal component analysis (PCA, varimax rotation) was used to determine the dietary patterns of the individuals included in the present study [22,32]. Dietary intakes obtained from the FFQ in portions per week from all 23 categories, both at baseline and at four weeks, were entered into the PCA. Components were retained based on the eigenvalue (>1), the inflection point of the scree plot, and interpretability. A food category was considered to contribute to a dietary pattern if the factor loading was <−0.2 or >0.2. Dietary pattern scores were calculated by summing the intakes of the contributing food categories weighted by their factor loadings. Higher dietary pattern scores represent higher intakes of positively associated food items and lower intakes of negatively associated food items. The patterns were labeled according to the food categories that were positively related to that dietary pattern. Dietary macronutrient composition (total caloric intake, carbohydrate, protein, fat, saturated fat, and fiber intake) was estimated based on the FFQ validation [22].

### 2.4. Analysis of Faecal Samples

Fecal sample analysis was performed by MyMicroZoo^TM^. The participants were provided with a sampling kit and the instructions for taking a fecal sample at home at baseline and after four weeks. The DNA was isolated using the ZymoBIOMICS 96 MagBead DNA Kit (Zymo Research, Irvine, CA, USA). The gDNA served as a template for the PCR amplification of part of 16S rRNA genes and subsequent analysis by next-generation sequencing using Illumina MiSeq. In short, amplicons of the V3-V4 regions of the 16S rRNA genes were generated by PCR with the primers 341F (5′-CCTACGGGNGGCWGCAG-3′) and 785R (5′-GACTACHVGGGTATCTAATCC-3), which generated a ~630 bp amplicon [33] complemented by standard Illumina adapters. The thermocycler (ThermoFisher) program used was the following: 30 s at 98 °C degrees, followed by 25 cycles of 10 s at 98 °C, 30 s at 55 °C, 30 s at 72 °C, then 5 min at 72 °C, and holding at 4 °C. Unique Index Primers were attached to the amplicons in each sample with a second PCR cycle with the following program: 30 s at 98 °C degrees, followed by 6 cycles of 10 s at 98 °C, 30 s at 55 °C, 30 s at 72 °C, then 5 min at 72 °C, and holding at 4 °C. The PCR products were purified using Agencourt© AMPure^®^ XP (Becker Coulter) and the DNA concentration was measured by fluorometric analysis (dsDNA HS kit, Quant-it, Invitrogen). Subsequently, PCR amplicons were equimolarly pooled, followed by sequencing on an Illumina MiSeq with the paired-end 300 cycles protocol and indexing. FASTQ read sequence files were generated using bcl2fastq version 2.20 (Illumina). The initial quality assessment was based on data passing the Illumina Chastity filtering. Subsequently, reads containing PhiX control signals were removed using an inhouse filtering protocol. In addition, reads containing (partial) adapters were clipped (up to a minimum read length of 50 bp). The second quality assessment was based on the remaining reads using the FASTQC quality control tool version 0.11.8.

The Illumina paired reads were merged into single reads (so-called pseudoreads) through sequence overlap, after the removal of the forward and reverse primers. Data were analyzed and demultiplexed based on sample-specific barcodes. Chimeric pseudoreads were removed using USEARCH 9.240 and the remaining reads were aligned to the RDP 16S rRNA gene database 11.5. Based on the alignment scores of the pseudoreads, the taxonomic depth of the lineage was based on the identity threshold of the rank; species, 99%, genus, 97%, family, 95%, order, 90%, class, 85%, and phylum, 80%.

### 2.5. Statistical Analysis

Differences in somatic and mental symptoms, physical activity, body weight, BMI, waist circumference, and dietary pattern scores between baseline and after week 4 were assessed with paired *t*-tests. The Shannon entropy of counts, a metric of microbiota α-diversity, was calculated using USEARCH 9.2 with operational taxonomic unit (OTU) clustering with a sequencing identity threshold of 97%, after subsampling from the entire set to account for different sampling depths. Bacterial species, genera, families, and phyla were used for analysis if the number of reads was 0.1% of the total amount of reads and if they were present in at least 10 samples. The Bray–Curtis dissimilarity metric was calculated with the genera selected with the aforementioned criteria. Single species, genera, families, and phyla were selected for analysis when the relative abundance across all samples was >1%. Associations between Bray–Curtis dissimilarities and dietary intake or other outcomes were tested using PERMANOVA with 1000 permutations.

Linear modeling was used to examine the correlation between dietary intake, dietary pattern scores or macronutrient composition, somatic and mental symptoms, body weight, and the gut microbiota. This was performed in three different ways. Firstly, changes in dietary intake or health outcomes were modeled against changes in the gut microbiota composition. Secondly dietary intake or health outcomes after four weeks of dietary intervention were modeled against the final gut microbiota composition. Lastly, final dietary intake was modeled against changes in the gut microbiota, and the final gut microbiota were modeled against changes in complaints. Previously, it was shown in the present study that endpoint variations in dietary intake were better correlated with health outcomes than changes in dietary intake [34]. Models were adjusted for baseline symptom scores, sex, age, changes in BMI, and changes in physical activity when applicable. Only participants with values for all confounding factors were included.

All statistical tests were 2-sided and significance was assumed when *p* ≤ 0.05. Benjamini–Hochberg correction was used for multiple testing. All statistical analysis was performed in R (version 4.0.3).

## 3. Results

### 3.1. Participants and Baseline Values

**Figure 1:**
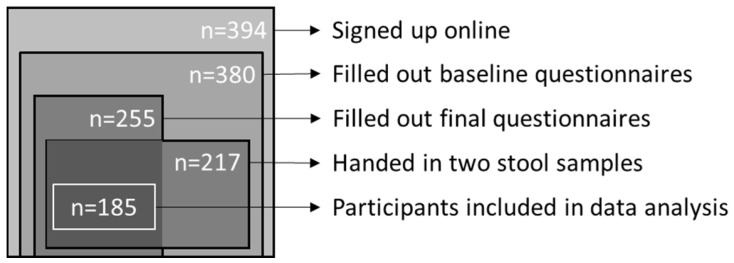
Flowchart visualizing the recruitment of participants.

**Figure 2:**
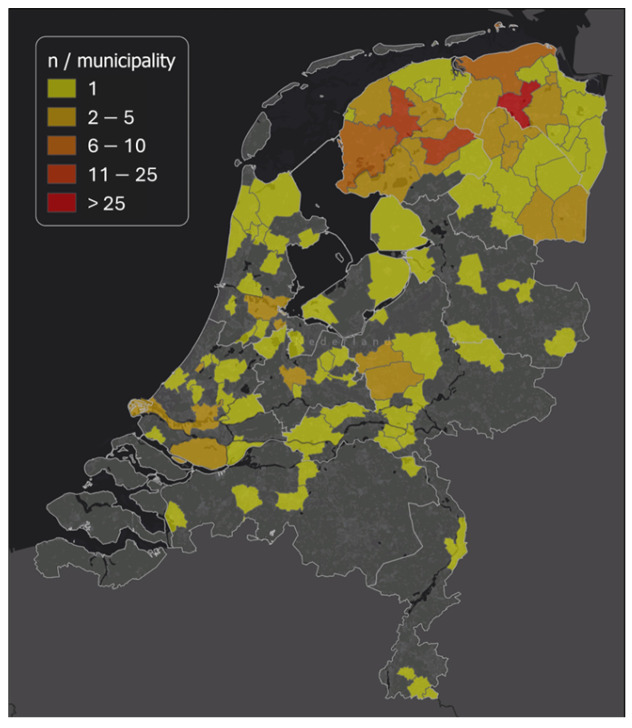
Geographical distribution of Dutch participants based on municipality.

Over the course of the study, there was a dropout rate of 53%, which was equally divided over sex, age, education level, BMI, and baseline mental and somatic symptoms. Completers (n = 185) filled out questionnaires at baseline and at week 4, and also handed in stool samples both at baseline and at four weeks (Figure 1). A few participants missed the week 4 questionnaire submission timeslot (i.e., because of closure of the website www.etenvoordewetenschap.nl, accessed on 27 August 2024). Upon verification of these participants’ continued adherence in week 4, week 3 questionnaire data were used instead. Completers were, on average, 49.1 ± 14.9 years old, 80.5% were female, and 70.3% had completed higher education (Table 1). In total, 72% of the participants lived in the north of the Netherlands (province Friesland, 34%, Groningen, 29%, and Drenthe, 9%) (Figure 2). The baseline mental symptom score was 14.7 ± 13.9 and baseline somatic symptom score was 7.5 ± 4.8.

### 3.2. Dietary Intake and Health Outcomes

The principal component analysis revealed five dietary patterns, which, in total, explained 42.0% (11.5%, 9.5%, 8.7%, 6.4%, and 5.8%, respectively) of the variance in dietary intake (Table 2). Positive loadings of food groups indicate that a food item is highly associated with the corresponding dietary pattern, while negative loadings indicate that there is an inverse correlation. The first dietary pattern, labeled “Processed foods”, was high in snacks, candy, cookies, sugar sweetened beverages, refined grain products, wine, pasta, rice, and potatoes and low in fruit. The second pattern, labeled “Animal source foods”, was high in fresh meat, eggs, fresh fish, processed meat products, and processed fish products and low in fruit, wholegrain products, pasta, rice, potatoes, and vegetarian meat replacements. The third pattern, labeled “Pescatarian”, was high in vegetables, nuts, water, fruit, processed fish products, vegetarian meat replacements, pasta, rice, potatoes, fresh fish, and candy, and was low in refined grain products and sugar sweetened beverages. The fourth pattern, labeled “Traditional Dutch”, was high in wholegrain products, dairy, tea, processed meat products, pasta, rice and potatoes, and low in gluten-free bread replacements. The last dietary pattern, labeled “Party”, had high intakes of coffee, alcohol, processed fish products, and wine, and low intakes of vegetables, tea, sugar sweetened beverages, and water.

**Figure 3:**
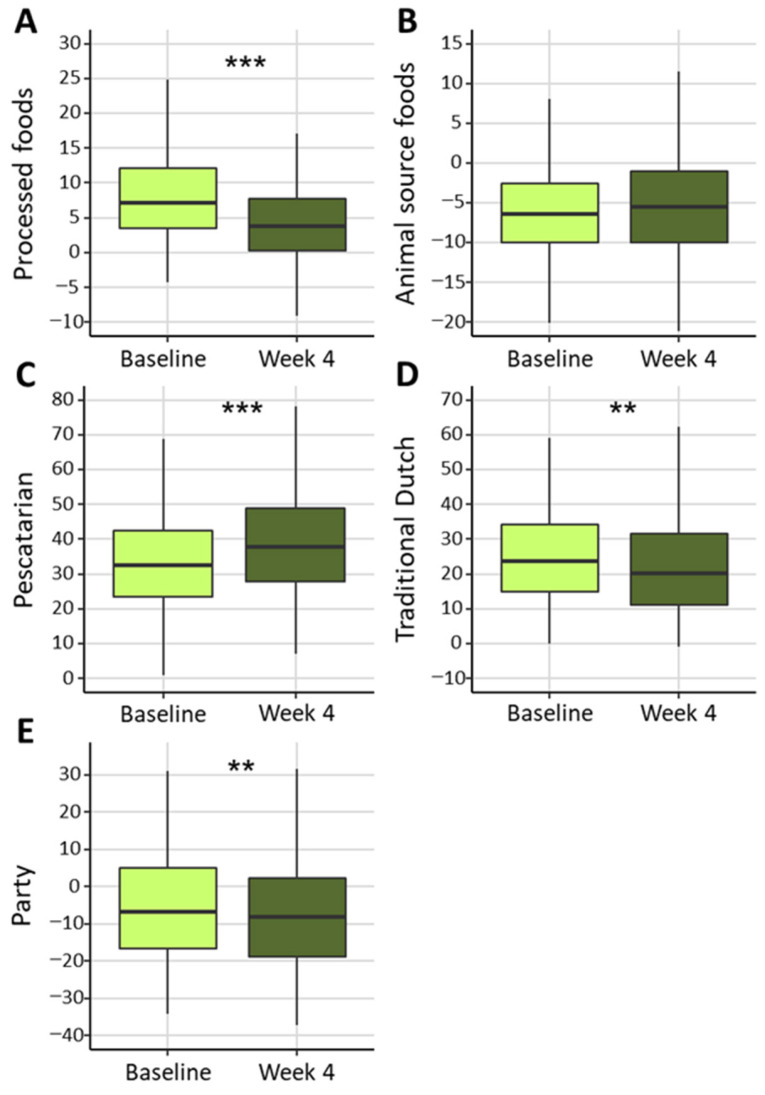
Dietary pattern scores at baseline and week 4 for (**A**) Processed foods, (**B**) Animal source foods, (**C**) Pescetarian, (**D**) Traditional Dutch, and (**E**) Party. ** *p* < 0.01 and *** *p* < 0.001.

Intake of the Processed foods, Traditional Dutch, and Party patterns was significantly decreased at week 4. Intake of the Pescatarian pattern was significantly increased at week 4, whereas intake of the Animal source foods pattern did not significantly change (Figure 3). Overall, there was a significant reduction in caloric intake and the energy percentage gained from carbohydrates, whereas there was a significant increase in the energy percentage gained from fat. There were decreases in the absolute intake of carbohydrates, fat, and protein (Table 3). The absolute intake of fiber, however, was unaltered at four weeks.

Next to the changes in dietary patterns and macronutrient intake, there were significant changes in health outcomes after four weeks of dietary intervention. A significant reduction in the number of mental and somatic symptoms was observed, as well as significant reductions in body weight, BMI, and waist circumference. Physical activity did not change (Table 4).

### 3.3. Gut Microbiota Composition

**Figure 4:**
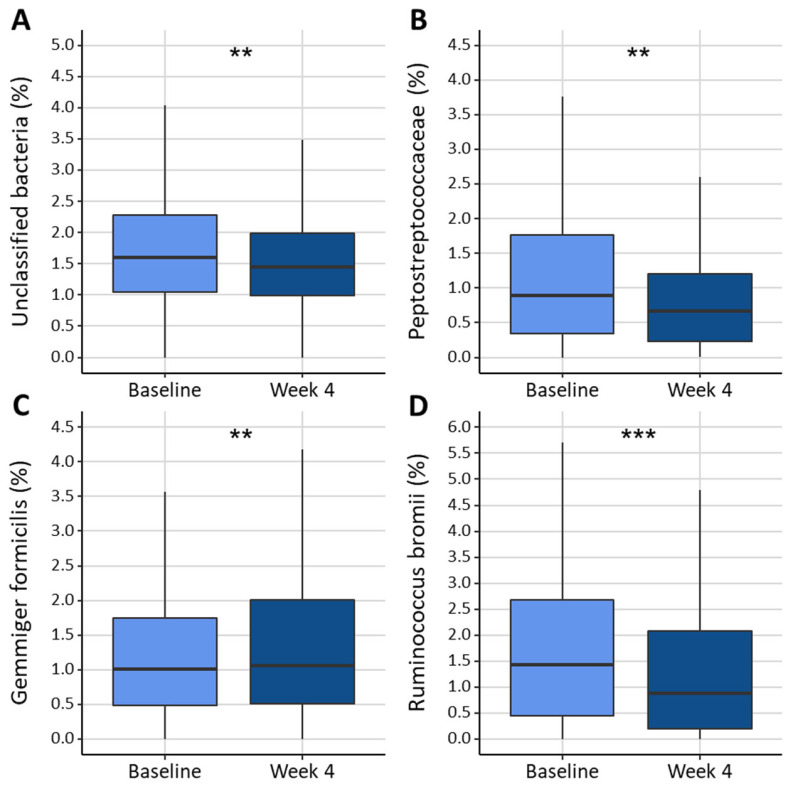
Bacterial abundance of (**A**) Unclassified bacteria (%), (**B**) *Peptostreptococcaceae* (%), (**C**) *Gemmiger formicilis* (%), and (**D**) *Rumminococcus bromii* (%). at baseline and at week 4, ** *p* < 0.01 and *** *p* < 0.001.

Based on their >1% bacterial abundance in the samples, we selected 6 phyla, 20 families, 29 genera, and 27 species (Supplemental Table S1). Significant changes in relative abundance were found for *Gemmiger formicilis* and *Ruminococcus bromii* (both within the family of Oscillospiraceae) after four weeks of dietary interventions, with an increase in the former and a decrease in the latter. On the family level, the relative abundance of *Peptostreptococcaceae* decreased, and on the phylum level, unclassified bacteria reduced significantly (Figure 4). There were no significant changes in the Shannon diversity index and Firmicutes/Bacteroidetes ratio.

### 3.4. Relation Between Dietary Intake and Gut Microbiota

Firstly, changes (i.e., from baseline to the end of the study) in the Shannon index were positively correlated with changes in the energy intake of fat (B = 0.065, Adj. r^2^ = 0.24, *p* = 0.04). However, there were no correlations between changes in individual bacteria and changes in dietary pattern scores or macronutrient intake.

Secondly, correlation analyses between the final gut microbiota composition and final dietary intakes were performed (see Table 5 below).

*Blautia obeum* abundance was positively correlated with the intake of Animal source foods and the percentual energy intake of fat and saturated fat, while it was negatively correlated with the percentual energy intake of carbohydrates. *Ruminococcus bromii* abundance was negatively correlated with the intake of Animal source foods and the percentual energy intake of fat and saturated fat. It was also positively correlated with the percentual and absolute energy intake of carbohydrates and fiber intake. *Gracilibacter thermotolerans* and *Flintibacter butyricus* abundances were positively correlated with the intake of Pescatarian foods, whereas *Bifidobacterium adolescentis* abundance was negatively correlated with Pescatarian intake. *Akkermansia muciniphila* was positively correlated with the percentual energy intake of saturated fat. Most of these results on the species level were also detected on the genus, family, and phylum levels, but could be traced back to these single bacterial species and are, therefore, not shown. On the genus level, *Prevotella* and *Roseburia* were negatively correlated with the percentual energy intake of saturated fat. On the family level, *Bacteroidaceae* and *Erysipelotrichaceae* were positively correlated with the percentual energy intake of fat and saturated fat intake. Lastly, *Clostridiaceae* was also positively correlated with the percentual energy intake of saturated fat (Table 5).

Bray–Curtis dissimilarity on the genus level was calculated for all samples at week 4. PERMANOVA analyses were performed to quantify the variance in the samples explained by dietary intake, symptoms, and body weight. At week 4, beta diversity was related to caloric intake (*p* < 0.01), the absolute intake of carbohydrates (*p* = 0.03), fats (*p* < 0.001), and proteins (*p* < 0.01), and BMI (*p* = 0.02). No relations were found with dietary patterns. Finally, also no correlations were found between the Firmicutes/Bacteroidetes ratio and dietary intake.

### 3.5. Relation of Health Outcomes and Gut Microbiota

Several intervention-induced changes in health parameters were found to be related to changes in the gut microbiota parameters. Firstly, a reduction in BMI was correlated with an increased abundance of the family *Erysipelotrichaceae*, however, this association was not very strong (B = −0.069, Adj. r^2^ = 0.10, *p* < 0.01). On the genus level, a reduction in waist circumference was correlated with a reduction in *Oscillibacter* (B = 0.625, Adj. r^2^ = 0.16, *p* < 0.01). No relations were found between changes in somatic symptoms and changes in the gut microbiota parameters. Much stronger correlations were found between changes in mental health outcomes and the gut microbiota parameters. In particular, a reduction in total mental symptoms was strongly correlated with an increase in *Ruminococcus bicirculans* abundance (B = −0.659, Adj. r^2^ = 0.64, *p* < 0.001). A reduction in stress symptoms was also correlated with a reduction in *Barnesiella intestinihominis* abundance (B = 0.111, Adj. r^2^ = 0.75, *p* < 0.001), but this correlation was no longer significant after correcting for dietary pattern scores (*p* = 0.10).

Also, the relation between endpoint health outcomes and the gut microbiota composition after four weeks of dietary intervention was investigated. BMI at four weeks was positively correlated with the species *E. hallii* and the genera *Clostridium* and *Coprococcus*, and negatively correlated with the families *Clostridiaceae*, *Erysipelotrichaceae*, *Lachnospiraceae*, and *Peptostreptococcaceae*. Waist circumference was positively correlated with the species *E. hallii* and *Eubacterium rectale*, the genera *Coprococcus*, and unclassified *Lachnospiraceae*, and negatively correlated with the genus *Clostridium* and the family *Clostridiaceae.* Lastly, there was a positive correlation between stress symptoms and the genus *Prevotella* (Table 6).

## 4. Discussion

Previously, we found that a self-initiated and freely chosen four-week dietary intervention adopted by ~280 completing participants led to reduced mental and somatic health complaints, as well as a reduction in body weight. A reduction in relative carbohydrate intake, reduced total energy intake, reduced intake of “Processed foods”, and increased intake of “Wheel of five” foods appeared to be key in these effects [22]. The current study was performed in a subset (n = 185) of those individuals who additionally provided fecal samples at baseline and at the end of the intervention, allowing us to potentially relate gut microbiota characteristics to dietary intake and the observed health benefits of the self-implemented and freely chosen diet. An important prerequisite for the current study was the ability to largely reconfirm the general health benefits in this smaller subset, again showing relative reductions in carbohydrate and total energy intake and a reduced intake from “Processed Foods” items to be related to reduced health complaints and reductions in body weight and BMI. The gut microbiota analysis in the current study showed that the four-week dietary intervention caused changes in the relative abundance of Eubacteriales bacteria, namely an increase in the *Gemmiger formicilis* species and reductions in the *Ruminococcus bromii* species and *Peptostreptococcaceae* family. These effects were not associated with changes in the Shannon diversity index or Firmicutes/Bacteroidetes ratio, indicating no major shifts and/or abundances/dysbiotic properties as a consequence of the dietary changes.

The Eubacteriales order belongs to the class of Clostridia and consists of species that are usually considered as normal commensal bacteria contributing to gut homeostasis. In particular, *Ruminococcaceae* (to which the *Gemmiger* and *Ruminococcus* species also belong) consists of bacteria that are able to digest the complex carbohydrates found in high-fiber foods [35,36], and some of which produce short-chain fatty acids (SCFAs) that are beneficial for gut health and beyond [37]. While *R. bromii* has received a lot of attention lately as one of the exponents of this class, its abundance decreased over the four-week intervention in the present study—probably as a result of the reduced carbohydrate intake—while the abundance of *G. formicilis* increased. *G. formicilis* is atypical in the sense that it has been related to type 2 diabetes mellitus [38], but there is actually also support for the fact that its abundance is increased in metabolically healthy obese versus metabolically non-healthy obese individuals [39]. In this light, it is very interesting that the *G. formicilis* abundance was reported to be lowered in individuals with irritable bowel disease (IBD; i.e., according to a meta-analysis including nine different IBD populations [40]), however, the precise causal mechanism and implications of this remain to be established. The reduction in *Peptostreptococcaceae* abundance is in line with publications linking its high abundance with an unhealthy dietary intake [41] and poor quality of life [42], although contrasting findings have also been mentioned [43]. Indeed, the general somatic and mental complaints of participants in the present study decreased, while the intake of “Processed foods” also decreased. Besides the latter, the intake of fat and protein also increased, as well as an increase in the intake from the “Pescatarian” food group. It may be hypothesized that these changes were somehow related to the changes observed in *G. formicilis* and *Peptostreptococcaceae*, either directly or via effects mediated by the host. In fact, lately, more studies have shown a positive impact of fat intake on the gut microbiota [44]. Although we did not observe related changes in fat or protein intake with the aforementioned bacteria, we did observe that the change in the Shannon index was positively correlated with the change in fat intake. Dietary fat includes, amongst others, mono- and polyunsaturated fats, as well as saturated fat, which all could have varied beneficial or deleterious effects on the gut microbiota composition too [45,46]. One of the bacteria that was positively correlated with the intake of saturated fat at the end of the dietary intervention was *Akkermansia muciniphila*. *A. muciniphila* has recently gained interest as probiotic for its beneficial effects on body weight, fat mass, and hip circumference [47,48]. Negative correlations between *A. muciniphila* abundance and overweight [49], obesity [50,51], untreated T2D [52], and hypertension have been reported [47,53,54].

Besides the overall shifts in Eubacterial abundances over the course of the intervention and the change in the Shannon index being positively correlated with the change in fat intake over the course of the four-week intervention study, we did not observe other changes in bacterial abundance that were related to changes in dietary intake, nor did we observe correlations between the baseline measurements of bacterial abundance/diversity and dietary intake patterns. Apparently, the variation between participants was too high to yield distinct relations before the start of the intervention. Analysis of the endpoint characteristics of the participants, however, did yield several relations. Our explanation for this phenomenon is that the variation between individuals became smaller over the course of the interventions (e.g., due to less snacking and lower intake of processed foods), despite the fact that the dietary interventions of participants were self-chosen. Indeed, at the end of study, we found several correlations between diet and bacterial abundance. For example, the above-discussed *R. bromii* abundance at the end of study was, as expected, correlated with endpoint carbohydrate and fiber intake. *Blautia obeum* (phylum Firmicutes and class Clostridia), reported for its ability to regulate host health and alleviate metabolic syndrome (MetS) [55], was negatively correlated with carbohydrate intake and was positively correlated with fat intake and the intake of the Animal source foods pattern. In our previous meta-analysis focusing on low-carbohydrate and low-fat diets and markers of MetS, we reported that lower carbohydrate and increased fat and protein intake were related to a decrease in markers of MetS [34]. It may be speculated that a higher intake of fat (not only from animal food) and lower intake of carbohydrates, each seen in the current study, may increase *B. obeum* and potentially also reduce the risk for developing NCDs.

Finally, we also investigated the relations between bacterial abundance and (subjective) somatic and mental health characteristics. First of all, the magnitude by which the BMI was reduced over the course of the intervention was significantly correlated with the extent to which the abundance of *Erysipelotrichaceae* was increased. An increased abundance of *Erysipelotrichaceae* has frequently been mentioned as pro-inflammatory and seems to be associated with obesity and lipid metabolism [56]. However, the same inverse correlation that we found in our study between BMI and *Erysipelotrichaceae* abundance was shown on a low-dose probiotic intervention, possibly indicating that the bacteria act synergistically with probiotics to lower body mass and BMI [57]. Furthermore, reductions in total mental symptoms were correlated strongly with an increase in *Ruminococcus bicirculans* abundance, a well-known utilizer of plant cell wall polysaccharides (including beta-glucans), but unlike *R. bromii*, they are unable to degrade resistant starches [35,58]. Reductions in stress symptoms were also correlated with a reduction in *Barnesiella intestinihominis* abundance, but this correlation was no longer significant after correcting for dietary pattern scores.

At the end of the study, *Eubacterium rectale* (phylum Firmicutes and class Clostridia, an SCFA producer [59]) and *Eubacterium hallii* were positively correlated with the endpoint BMI and waist circumference. In obese individuals, a reduction in carbohydrate intake has been shown to cause a reduction in *E. rectale* [60], and increased *E. rectale* abundance has previously been linked to a higher intake of (ultra-)processed foods [61,62]. This complements our findings with regard to the relation between reductions in body weight and intake of processed foods and carbohydrates [22]. A higher *Prevotella* abundance was found to be correlated with higher stress scores, which is interesting, since *Prevotella* is associated with a higher fiber and vegetable intake [11], which, in general, are related to a better mental health [63].

This study has some limitations. Especially for their effects on the gut microbiota, microbiota-accessible carbohydrates in food items are of importance [64]. A more specified analysis of dietary intake and macronutrient content would be of interest, and in particular, information on simple sugars versus carbohydrates and the type of fat intake would provide further insights into the specific relations between the gut microbiota and diet composition. Secondly, we were not able to perform a functional analysis of the gut microbiota, but this would have given further insights into the functional changes occurring in the gut, which may be more related to the health outcomes we observed. We used 16S data for analyses, which are less reliable on the species than on the genus level. However, many of the correlations we found on the species level were also present on higher levels, but could be traced back to that single species, indicating that the correlations found were reliable. Lastly, a potential limitation of this study refers to the credibility of self-reported anthropometric measures (including body weight and waist circumference). We tried to optimize this as much as possible by carefully explaining to the participants at the beginning of the study the importance of the reliability of their data, and how they should collect and report them in a standardized and equitable manner, no matter the outcome. Thus, although mistakes can never fully be ruled out in this or any other study, we are confident that the vast majority of our data are credible and that our conclusions are justified.

## 5. Conclusions

We conclude that a self-initiated four-week dietary intervention improved mental and somatic health and induced changes in the gut microbiota composition, with an increased fat intake and reduced carbohydrate and “Processed foods” intake probably being the most prominent steering components. A number of individual bacterial taxa were correlated with macronutrient intake, dietary patterns, BMI, and mental health, with the most profound contribution being that of the *Eubacteriales* family to the microbiota–gut–brain axis. An intriguing issue, however, is fact that the most prominently “overall” changed bacterial species, i.e., *Gemmiger formicilis*, *Ruminococcus bromii*, and *Peptostreptococcaceae* (with the abundance of the former increasing and the latter two decreasing over the four-week dietary intervention) were, except for *Ruminococcus bromii*, neither linearly related to specific diet intake parameters nor to specific somatic and mental health indices. At the moment, we do not know the relevance of these overall changes in light of the various bacterial species, classes, etc., that were observed to be linearly related to dietary parameters and/or health parameters, particularly at the end of the four-week intervention, but did not stand out to be generally altered. The exponent observation of the latter was the increase in *R. bicirculans* being strongly individually correlated with reductions in mental complaints. These results may be interpreted to indicate that overall changed—versus individually correlated—alterations in microbiota abundances and their relations with host health characteristics represent different levels of regulation, which remain to be further untangled.

## Figures and Tables

**Table 1:** Baseline demographic, behavioral, and clinical characteristics of study participants.

	Mean (SD)
Age (y)	49.1 (14.9)
Gender, female (%)	80.5
High education (%)	70.3
Smokers, current (%)	4.3
Diagnosed with depression (%)	7.0
High/moderate scores depression (%)	8.1
High/moderate scores anxiety (%)	10.8
High/moderate scores stress (%)	2.2
Probiotic use in the last 6 months (%)	9.7
Antibiotic use in the last 6 months (%)	21.1
High blood pressure diagnosis (%)	8.1
Diabetes Type 2 diagnosis (%)	2.7
High cholesterol diagnosis (%)	7.6

**Table 2:** Dietary patterns identified by means of PCA and the factor loadings per food category.

Factor Loadings of Food Categories in Identified Dietary Patterns									
Processed Foods		Animal Source Foods		Pescatarian		Traditional Dutch		Party	
Snacks	0.754	Fresh meat	0.672	Vegetables	0.698	Wholegrain products	0.694	Coffee	0.732
Candy	0.697	Eggs	0.565	Nuts	0.696	Dairy	0.545	Alcohol	0.551
Cookies	0.648	Fresh fish	0.543	Water	0.490	Tea	0.523	Processed fish products	0.341
Sugar sweetened beverages	0.474	Processed meat products	0.412	Fruit	0.432	Processed meat products	0.407	Wine	0.336
Refined grain products	0.426	Processed fish products	0.363	Processed fish products	0.373	Pasta, rice, and potatoes	0.320	Vegetables	−0.219
Wine	0.239	Fruit	−0.303	Vegetarian meat replacements	0.294	Gluten-free bread replacements	−0.471	Tea	−0.229
Pasta, rice, and potatoes	0.210	Wholegrain products	−0.303	Pasta, rice and potatoes	0.240			Sugar sweetened beverages	−0.242
Fruit	−0.201	Pasta, rice, and potatoes	−0.326	Fresh fish	0.237			Water	−0.407
		Vegetarian meat replacements	−0.549	Candy	0.210				
				Refined grain products	−0.273				
				Sugar sweetened beverages	−0.288				

**Table 3:** Dietary macronutrient composition at baseline and at week 4.

	Baseline	Week 4	*p* ^a^
Caloric intake (kcal)	1798.3 (259.4)	1733.3 (266.8)	<0.001
Carbohydrate intake (en%)	41.4 (3.6)	40.4 (3.5)	<0.001
Fat intake (en%)	35.7 (1.6)	36.0 (1.9)	0.015
Protein intake (en%)	17.0 (1.6)	17.1 (1.5)	0.096
Saturated fat intake (en%)	12.5 (0.8)	12.5 (0.9)	0.375
Fiber intake (g)	21.8 (2.9)	21.6 (3.3)	0.378
Carbohydrate intake (g)	185.9 (29.9)	174.9 (30.2)	<0.001
Fat intake (g)	76.8 (16.7)	74.7 (16.3)	<0.001
Protein intake (g)	71.3 (9.8)	69.2 (9.8)	<0.001

en% = energy percentage, ^a^ Difference between baseline and week 4.

**Table 4:** Outcomes at baseline and at week 4.

	n	Baseline	n	Week 4	*p* ^a^
Weight (kg)	175	74.2 (14.0)	174	73.7 (14.1)	<0.001
BMI (kg/m^2^)	175	24.5 (4.3)	172	24.3 (4.3)	<0.001
Waist circumference (cm)	174	89.3 (12.9)	178	88.7 (13.3)	<0.001
DASS score	185	14.7 (13.9)	185	6.6 (8.7)	<0.001
Depression score	185	5.2 (5.2)	185	2.3 (3.0)	<0.001
Anxiety score	185	4.1 (4.5)	185	2.1 (3.1)	<0.001
Stress score	185	5.2 (5.0)	185	2.2 (3.2)	<0.001
PHQ-15 score	185	7.5 (4.8)	185	4.8 (3.9)	<0.001
Activity pas-2 (METs/24 h)	180	41.0 (5.2)	177	40.9 (4.8)	0.791
Shannon index	185	5.2 (0.7)	185	5.2 (0.7)	0.422
Firmicutes/Bacteroidetes ratio	185	3.3 (1.7)	185	4.6 (15.8)	0.251

^a^ Difference between baseline and after week 4.

**Table 5:** Correlations of final gut microbiota abundance and final dietary intake.

	B	SE	Adj. r^2^	*p*
**Species**				
*Blautia obeum*				
Animal source foods	0.035	0.012	0.04	0.004
Carbohydrate intake (en%)	−0.092	0.028	0.05	0.001
Fat intake (en%)	0.145	0.052	0.04	0.005
Saturated fat intake (en%)	0.030	0.107	0.04	0.006
*Ruminococcus bromii*				
Animal source foods	−0.067	0.018	0.06	<0.001
Carbohydrate intake (en%)	0.159	0.044	0.06	<0.001
Fat intake (en%)	−0.280	0.079	0.06	0.001
Saturated fat intake (en%)	−0.476	0.166	0.03	0.005
Fiber intake	0.181	0.044	0.08	<0.001
Carbohydrate intake (g)	0.021	0.006	0.06	<0.001
*Bifidobacterium adolescentis*				
Pescatarian	−0.058	0.017	0.05	0.001
*Gracilibacter thermotolerans*				
Pescatarian	0.039	0.013	0.07	0.003
*Flintibacter butyricus*				
Pescatarian	0.021	0.006	0.05	0.001
*Akkermansia muciniphila*				
Saturated fat intake (en%)	0.591	0.196	0.06	0.003
**Genus**				
*Prevotella*				
Saturated fat intake (en%)	−1.278	0.408	0.07	0.002
*Roseburia*				
Saturated fat intake (en%)	−0.478	0.147	0.06	0.001
**Family**				
*Bacteroidaceae*				
Fat intake (en%)	0.709	0.268	0.12	0.009
Saturated fat intake (en%)	1.514	0.554	0.13	0.007
*Erysipelotrichaceae*				
Fat intake (en%)	0.288	0.096	0.10	0.003
Saturated fat intake (en%)	0.674	0.198	0.11	<0.001
*Clostridiaceae*				
Saturated fat intake (en%)	0.583	0.234	0.15	0.014

en% = energy percentage, B = Unstandardized beta, SE = standard error, and Adj. r^2^ = adjusted r^2^.

**Table 6:** Correlations between final health outcomes and final gut microbiota composition.

	B	SE	Adj. r^2^	*p*
**Species**				
BMI				
*Eubacterium hallii*	1.470	0.336	0.14	<0.001
Waist circumference				
*Eubacterium hallii*	3.117	1.038	0.19	0.003
*Eubacterium rectale*	1.603	0.480	0.20	0.001
**Genus**				
Stress symptoms				
*Prevotella*	0.176	0.051	0.12	<0.001
BMI				
*Clostridium*	−0.532	0.114	0.15	<0.001
*Coprococcus*	0.625	0.168	0.11	<0.001
Waist circumference				
*Clostridium*	−1.182	0.353	0.20	0.001
*Coprococcus*	1.743	0.506	0.20	<0.001
*Unclassified Lachnospiraceae*	1.630	0.480	0.20	<0.001
**Family**				
BMI				
*Clostridiaceae*	−0.542	0.111	0.16	<0.001
*Erysipelotrichaceae*	−0.465	0.138	0.10	<0.001
*Lachnospiraceae*	0.103	0.036	0.08	0.005
*Peptostreptococcaceae*	−0.522	0.143	0.11	<0.001
Waist circumference				
*Clostridiaceae*	−1.266	0.343	0.21	<0.001

B = Unstandardized beta, SE = standard error, and Adj. r^2^ = adjusted r^2^.

## Data Availability

The data presented in this study are available on request from the corresponding author due to privacy restrictions.

## Supplementary Materials

The following supporting information can be downloaded at: https://www.mdpi.com/article/10.3390/nu16203544/s1, Table S1: Selected species for beta diversity calculation with their corresponding genus, family and phylum. Highlighted species, genera, families, and phyla were selected for in-depth analysis.
