# Supplementary material for: Self-Initiated Dietary Adjustments Alter Microbiota Abundances: Implications for Perceived Health
Source: Nutrients. 2024 Oct 18;16(20):3544. doi: 10.3390/nu16203544 (PMC11510366; doi:10.3390/nu16203544)
Supplement: Supplementary file 1 [file nutrients-16-03544-s001.zip › nutrients-3204635-supplementary.pdf]

Table S1: Selected species for beta diversity calculation with their corresponding genus, family and phylum. Grey-colored species, genera, families and phyla were selected for in-depth analysis.

Phylum	Family	Genus	Species
Actinobacteria	Bifidobacteriaceae	Bifidobacterium	Bifidobacterium adolescentis
Actinobacteria	Bifidobacteriaceae	Bifidobacterium	Bifidobacterium bifidum
Actinobacteria	Bifidobacteriaceae	Bifidobacterium	Bifidobacterium longum
Actinobacteria	Bifidobacteriaceae	Bifidobacterium	Bifidobacterium pseudocatenulatum
Actinobacteria	Coriobacteriaceae	Collinsella	Collinsella aerofaciens
Actinobacteria	Coriobacteriaceae	Senegalimassilia	Senegalimassilia anaerobia
Actinobacteria	Eggerthellaceae	Adlercreutzia	Adlercreutzia equolifaciens
Actinobacteria	Eggerthellaceae	Denitrobacterium	Denitrobacterium detoxificans
Actinobacteria	Eggerthellaceae	Enterorhabdus	Enterorhabdus mucosicola
Bacteroidetes	Bacteroidaceae	Bacteroides	Bacteroides caccae
Bacteroidetes	Bacteroidaceae	Bacteroides	Bacteroides cellulosilyticus
Bacteroidetes	Bacteroidaceae	Bacteroides	Bacteroides coprocola
Bacteroidetes	Bacteroidaceae	Bacteroides	Bacteroides dorei
Bacteroidetes	Bacteroidaceae	Bacteroides	Bacteroides eggerthii
Bacteroidetes	Bacteroidaceae	Bacteroides	Bacteroides faecis
Bacteroidetes	Bacteroidaceae	Bacteroides	Bacteroides finegoldii
Bacteroidetes	Bacteroidaceae	Bacteroides	Bacteroides fragilis
Bacteroidetes	Bacteroidaceae	Bacteroides	Bacteroides galacturonicus
Bacteroidetes	Bacteroidaceae	Bacteroides	Bacteroides intestinalis
Bacteroidetes	Bacteroidaceae	Bacteroides	Bacteroides massiliensis
Bacteroidetes	Bacteroidaceae	Bacteroides	Bacteroides ovatus
Bacteroidetes	Bacteroidaceae	Bacteroides	Bacteroides plebeius
Bacteroidetes	Bacteroidaceae	Bacteroides	Bacteroides stercoris
Bacteroidetes	Bacteroidaceae	Bacteroides	Bacteroides thetaiotaomicron
Bacteroidetes	Bacteroidaceae	Bacteroides	Bacteroides uniformis
Bacteroidetes	Bacteroidaceae	Bacteroides	Bacteroides vulgatus
Bacteroidetes	Bacteroidaceae	Bacteroides	Bacteroides xylanisolvens
Bacteroidetes	Odoribacteraceae	Odoribacter	Odoribacter splanchnicus
Bacteroidetes	Porphyromonadaceae	Barnesiella	Barnesiella intestinihominis
Bacteroidetes	Porphyromonadaceae	Barnesiella	Barnesiella viscericola
Bacteroidetes	Porphyromonadaceae	Barnesiella	unclassified Barnesiella
Bacteroidetes	Porphyromonadaceae	Coprobacter	Coprobacter fastidiosus
Bacteroidetes	Porphyromonadaceae	Parabacteroides	Parabacteroides distasonis
Bacteroidetes	Porphyromonadaceae	Parabacteroides	Parabacteroides merdae
Bacteroidetes	Porphyromonadaceae	Tannerella	unclassified Tannerella
Bacteroidetes	Prevotellaceae	Alloprevotella	Alloprevotella rava
Bacteroidetes	Prevotellaceae	Paraprevotella	Paraprevotella clara
Bacteroidetes	Prevotellaceae	Prevotella	Prevotella copri
Bacteroidetes	Prevotellaceae	Prevotella	Prevotella dentalis
Bacteroidetes	Prevotellaceae	Prevotella	Prevotella oralis

Bacteroidetes	Prevotellaceae	Prevotella	Prevotella oris
Bacteroidetes	Prevotellaceae	Prevotella	Prevotella ruminicola
Bacteroidetes	Prevotellaceae	Prevotella	Prevotella stercorea
Bacteroidetes	Rikenellaceae	Alistipes	Alistipes finegoldii
Bacteroidetes	Rikenellaceae	Alistipes	Alistipes obesi
Bacteroidetes	Rikenellaceae	Alistipes	Alistipes onderdonkii
Bacteroidetes	Rikenellaceae	Alistipes	Alistipes putredinis
Bacteroidetes	Rikenellaceae	Alistipes	Alistipes senegalensis
Bacteroidetes	Rikenellaceae	Alistipes	Alistipes shahii
Bacteroidetes	Rikenellaceae	Rikenella	unclassified Rikenella
Bacteroidetes	unclassified Bacteroidales	unclassified Bacteroidales	unclassified Bacteroidales
Cyanobacteria	unclassified Cyanobacteria	unclassified Cyanobacteria	cyanobacterium enrichment
Euryarchaeota	Methanobacteriaceae	Methanobrevibacter	Methanobrevibacter smithii
Firmicutes	Acidaminococcaceae	Phascolarctobacterium	Phascolarctobacterium faecium
Firmicutes	Acidaminococcaceae	Phascolarctobacterium	Phascolarctobacterium succinatutens
Firmicutes	Christensenellaceae	Christensenella	Christensenella minuta
Firmicutes	Clostridiaceae	Butyricicoccus	Butyricicoccus pullicaecorum
Firmicutes	Clostridiaceae	Caloramator	Caloramator fervidus
Firmicutes	Clostridiaceae	Clostridium	Clostridium celatum
Firmicutes	Clostridiaceae	Clostridium	Clostridium cellulovorans
Firmicutes	Clostridiaceae	Clostridium	Clostridium chartatabidum
Firmicutes	Clostridiaceae	Clostridium	Clostridium phoceensis
Firmicutes	Clostridiaceae	Clostridium	unclassified Clostridium
Firmicutes	Clostridiaceae	Hungatella	Hungatella hathewayi
Firmicutes	Clostridiaceae	Lutispora	Lutispora thermophila
Firmicutes	Defluviitaleaceae	Vallitalea	Vallitalea pronyensis
Firmicutes	Erysipelotrichaceae	Catenibacterium	Catenibacterium mitsuokai
Firmicutes	Erysipelotrichaceae	Erysipelatoclostridium	Clostridium spiroforme
Firmicutes	Erysipelotrichaceae	Holdemanella	Holdemanella biformis
Firmicutes	Erysipelotrichaceae	Turicibacter	Turicibacter sanguinis
Firmicutes	Erysipelotrichaceae	Turicibacter	unclassified Turicibacter
Firmicutes	Erysipelotrichaceae	unclassified Erysipelotrichaceae	unclassified Erysipelotrichaceae
Firmicutes	Eubacteriaceae	Eubacterium	Eubacterium coprostanoligenes
Firmicutes	Eubacteriaceae	Eubacterium	Eubacterium desmolans
Firmicutes	Eubacteriaceae	Eubacterium	Eubacterium eligens
Firmicutes	Eubacteriaceae	Eubacterium	Eubacterium hallii
Firmicutes	Eubacteriaceae	Eubacterium	Eubacterium ramulus
Firmicutes	Eubacteriaceae	Eubacterium	Eubacterium ruminantium
Firmicutes	Gracilibacteraceae	Gracilibacter	Gracilibacter thermotolerans
Firmicutes	Lachnospiraceae	Anaerobium	Anaerobium acetethylicum
Firmicutes	Lachnospiraceae	Anaerostipes	Anaerostipes hadrus
Firmicutes	Lachnospiraceae	Anaerotaenia	Anaerotaenia torta
Firmicutes	Lachnospiraceae	Blautia	Blautia faecis

Firmicutes	Lachnospiraceae	Blautia	Blautia glucerasea
Firmicutes	Lachnospiraceae	Blautia	Blautia luti
Firmicutes	Lachnospiraceae	Blautia	Blautia massiliensis
Firmicutes	Lachnospiraceae	Blautia	Blautia obeum
Firmicutes	Lachnospiraceae	Blautia	Blautia stercoris
Firmicutes	Lachnospiraceae	Blautia	Blautia wexlerae
Firmicutes	Lachnospiraceae	Blautia	Ruminococcus torques
Firmicutes	Lachnospiraceae	Butyrivibrio	Butyrivibrio crossotus
Firmicutes	Lachnospiraceae	Coprococcus	Coprococcus catus
Firmicutes	Lachnospiraceae	Coprococcus	Coprococcus comes
Firmicutes	Lachnospiraceae	Coprococcus	Coprococcus eutactus
Firmicutes	Lachnospiraceae	Dorea	Candidatus Dorea
Firmicutes	Lachnospiraceae	Dorea	Dorea formicigenerans
Firmicutes	Lachnospiraceae	Dorea	Dorea longicatena
Firmicutes	Lachnospiraceae	Fusicatenibacter	Fusicatenibacter saccharivorans
Firmicutes	Lachnospiraceae	Herbinix	Herbinix luporum
Firmicutes	Lachnospiraceae	Hespellia	Hespellia porcina
Firmicutes	Lachnospiraceae	Lachnobacterium	Lachnobacterium bovis
Firmicutes	Lachnospiraceae	Lachnoclostridium	Clostridium aerotolerans
Firmicutes	Lachnospiraceae	Lachnoclostridium	Clostridium aldenense
Firmicutes	Lachnospiraceae	Lachnoclostridium	Clostridium clostridioforme
Firmicutes	Lachnospiraceae	Lachnoclostridium	Clostridium polysaccharolyticum
Firmicutes	Lachnospiraceae	Lachnoclostridium	Clostridium saccharolyticum
Firmicutes	Lachnospiraceae	Lachnoclostridium	Clostridium xylanolyticum
Firmicutes	Lachnospiraceae	Lachnospira	Lachnospira pectinoschiza
Firmicutes	Lachnospiraceae	Mobilitalea	Mobilitalea sibirica
Firmicutes	Lachnospiraceae	Murimonas	Murimonas intestini
Firmicutes	Lachnospiraceae	Roseburia	Roseburia faecis
Firmicutes	Lachnospiraceae	Roseburia	Roseburia hominis
Firmicutes	Lachnospiraceae	Roseburia	Roseburia intestinalis
Firmicutes	Lachnospiraceae	Roseburia	Roseburia inulinivorans
Firmicutes	Lachnospiraceae	unclassified Lachnospiraceae	Eubacterium rectale
Firmicutes	Lactobacillaceae	Lactobacillus	Lactobacillus rogosae
Firmicutes	Oscillospiraceae	Oscillibacter	Oscillibacter ruminantium
Firmicutes	Oscillospiraceae	Oscillibacter	Oscillibacter valericigenes
Firmicutes	Peptostreptococcaceae	Intestinibacter	Intestinibacter bartlettii
Firmicutes	Peptostreptococcaceae	Romboutsia	Romboutsia sedimentorum
Firmicutes	Peptostreptococcaceae	Terrisporobacter	Terrisporobacter glycolicus
Firmicutes	Ruminococcaceae	Acetanaerobacterium	Acetanaerobacterium elongatum
Firmicutes	Ruminococcaceae	Acetivibrio	Acetivibrio ethanolgignens
Firmicutes	Ruminococcaceae	Anaerobacterium	Anaerobacterium chartisolvens
Firmicutes	Ruminococcaceae	Anaeromassilibacillus	Anaeromassilibacillus senegalensis
Firmicutes	Ruminococcaceae	Candidatus Soleaferrea	Candidatus Soleaferrea

Firmicutes	Ruminococcaceae	Faecalibacterium	Faecalibacterium prausnitzii
Firmicutes	Ruminococcaceae	Gemmiger	Gemmiger formicilis
Firmicutes	Ruminococcaceae	Oscillospira	Oscillospira guilliermondii
Firmicutes	Ruminococcaceae	Ruminiclostridium	Clostridium leptum
Firmicutes	Ruminococcaceae	Ruminiclostridium	Clostridium methylpentosum
Firmicutes	Ruminococcaceae	Ruminiclostridium	Eubacterium siraeum
Firmicutes	Ruminococcaceae	Ruminiclostridium	Ruminiclostridium thermocellum
Firmicutes	Ruminococcaceae	Ruminococcus	Ruminococcus bicirculans
Firmicutes	Ruminococcaceae	Ruminococcus	Ruminococcus bromii
Firmicutes	Ruminococcaceae	Ruminococcus	Ruminococcus callidus
Firmicutes	Ruminococcaceae	Ruminococcus	Ruminococcus faecis
Firmicutes	Ruminococcaceae	Ruminococcus	Ruminococcus flavefaciens
Firmicutes	Ruminococcaceae	Ruminococcus	Ruminococcus lactaris
Firmicutes	Ruminococcaceae	Saccharofermentans	Saccharofermentans acetigenes
Firmicutes	Ruminococcaceae	Sporobacter	Sporobacter termitidis
Firmicutes	Streptococcaceae	Streptococcus	Streptococcus thermophilus
Firmicutes	unclassified Clostridiales	Flavonifractor	Flavonifractor plautii
Firmicutes	unclassified Clostridiales	Flintibacter	Flintibacter butyricus
Firmicutes	unclassified Clostridiales	Intestinimonas	Intestinimonas butyriciproducens
Firmicutes	unclassified Clostridiales	Intestinimonas	Intestinimonas massiliensis
Firmicutes	unclassified Clostridiales	Intestinimonas	Intestinimonas timonensis
Firmicutes	unclassified Clostridiales	Natranaerovirga	Natranaerovirga pectinivora
Firmicutes	unclassified Clostridiales	unclassified Clostridiales	Bacteroides pectinophilus
Firmicutes	Veillonellaceae	Dialister	Dialister invisus
Firmicutes	Veillonellaceae	Dialister	Dialister succinatiphilus
Proteobacteria	Desulfovibrionaceae	Bilophila	Bilophila wadsworthia
Proteobacteria	Desulfovibrionaceae	Desulfovibrio	Desulfovibrio piger
Proteobacteria	Enterobacteriaceae	Escherichia	Escherichia coli
Proteobacteria	Sutterellaceae	Parasutterella	Parasutterella excrementihominis
Proteobacteria	Sutterellaceae	Sutterella	Sutterella stercoricanis
Proteobacteria	Sutterellaceae	Sutterella	Sutterella wadsworthensis
Proteobacteria	unclassified Alphaproteobacteria	unclassified Alphaproteobacteria	alpha proteobacterium
Tenericutes	Anaeroplasmataceae	Anaeroplasma	Anaeroplasma bactoclasticum
Unclassfied	Unclassified	Unclassified	Unclassified
Verrucomicrobia	Akkermansiaceae	Akkermansia	Akkermansia muciniphila
